# Feeding the Young

**Published:** 1891-02

**Authors:** O. H. Phelps

**Affiliations:** Blocksbury, Cal.


### Feeding the Young.

BY O. H. PHELPS, M D., OF BLOCKSBURY, CAL.

It goes without saying that a large majority of the children who die under the age of 5 years perish from diseases of the alimentary canal, and in large cities the rate of mortality is such that even Maltheus would doubtles call a halt. That other causes than bad feeding come in for a share in the work of fatality we will not deny, neither will we discuss them in this paper. That bad feeding is the principal cause is fully borne out by the writer's observation and experience.

What is bad feeding? Mother's milk when the mother herself is badly fed all through the period of gestation and lactation; when her alimentary canal contains more or less fermenting material from access of undigested hydrocarbons; and her whole dietary lacks tissue building material to fairly support her own body, much less that of her child. A hungry man is quarrelsome and discontented—see the Irish people fed on potatoes. An under-fed mother is weak, nervous and fretful, and the effect of this alone is often disasterous to her child. The writer recalls a case where the child several times nearly perished in convulsions caused by an explosion of anger in the mother who was badly fed, weak and nervous. He advised weaning the child and all trouble ceased.

We will not discuss the proper feeding of the mother here, but would refer to a little book by Dr. E. Cutter, of New York, "Food in Motherhood," which we heartily commend for its treatment on this important subject.

A very common practice prevails among all classes of people of feeding solid food to infants 2 months old and upward. Sometimes they commence younger. That the digestive organs are not sufficiently developed to digest solid food is indicated by the lack of teeth to masticate with. And this may be taken as a safe guide as to the proper time to commence giving solid food. And if the child is bottle fed, sugar is usually added to the milk to assist in the alcoholic and acetic acid fermentation, and as a logical sequence of such outrageous feeding nature throws up the sponge and the doctor is called who often has to follow suit. Emaciation, colic, acrid dejections passing through the bowels, a burning seething liquid mass, only a little less irritating than molten lead.

How shall we feed to avoid all this?

First, don't give mother's milk, if it is bad milk. Don't ruin the child's digestion by persistent nursing when it is evident the mother can not furnish good milk. Don't give impure cow's milk, and good or bad, don't put sugar in it. Don't put starchy food in the child's—shall I say yeast pan?—unless you want more yeast, more colic, more cholera infantum, more dead babies.

Pure cow's milk sterilized will usually be well borne, if the digestion has not previously been ruined. This failing give beef essence and pepsin.

The writer has usually had indifferent success with the so-called infant foods of the shops. A child that can digest the best of them can as well or better digest a gruel, made of milk slightly thickened with meal made from twice baked bread. The bread made of entire wheat flour, is best when it comes with the advantage of cheapness and known purity, which can not always be said of commercially prepared infant foods. There are some nice formulas for taking out a portion of the caseine from cow's milk and otherwise making it as near like mother's milk as possible all of which are good on paper, but often very impracticable to be carried out, among the majority of the clientele of the busy practitioner. Some cases from practice will illustrate some of the results of bad feeding and their treatment. (Cases were here related.)

These will suffice to illustrate some of the conditions brought on by bad feeding. Very little medicine is needed in most cases. When indicated pepsin and salicin may be given. It is also desirable to flush the colon with warm water frequently.

The proper food is milk up two or three years of age, after that bread made from entire wheat flour (fine wheat flour is deprived of most of the tissue building material contained in wheat,) milk, eggs, lean beef and mutton, fruit without sugar; in fact sugar not at all.

Such a course will give less work to the undertaker, dentist, and doctor.—*Journal American Med. Ass'n.*

